# Behavioral Indicators of Heat Stress in Dairy Cows Under Subtropical Conditions: Comparison of Milking Systems

**DOI:** 10.3390/ani16111665

**Published:** 2026-05-29

**Authors:** Chun-Hsuan Chao, Kai-Chen Hsu, Chau-Hwa Chi

**Affiliations:** 1 Northern Region Branch, Taiwan Livestock Research Institute, Ministry of Agriculture, No. 207-5, Bitoumian, Xihu Township, Miaoli County 368003, Taiwan; 2 Department of Veterinary Medicine, National Taiwan University, No. 1, Sec. 4, Roosevelt Road, Taipei City 106216, Taiwan

**Keywords:** automated milking systems, panting behavior, behavioral variability, cross-correlation, generalized additive model, subtropical climate

## Abstract

Understanding behavioral responses of dairy cows to thermal load under different milking-system environments is essential for welfare monitoring in subtropical production systems, yet field-based evidence remains limited. This study compared heat-related behavioral responses between an automated milking system (AMS) and a conventional parlor system under commercial subtropical conditions, with the hypothesis that cows in the AMS would exhibit more stable and less variable behavioral patterns under heat stress. Panting, feeding, and rumination were continuously monitored and analyzed at the barn level in relation to the temperature–humidity index (THI). The AMS barn showed lower panting duration and reduced day-to-day variability, whereas the traditional barn exhibited higher panting levels and greater short-term fluctuations. Panting increased consistently with THI, showing a clear temporal trend (β ≈ 0.65 min/day), and emerged as the most sensitive indicator of thermal load, while feeding and rumination showed weaker responses. During heatwaves, panting increased markedly in both systems, with limited changes in feeding and rumination. Multivariate analyses (PCA) showed more compact clustering in the AMS barn, with higher variance explained along the first principal component (PC1 ≈ 44%), compared to greater dispersion in the traditional barn. These findings demonstrate that cows managed in the AMS exhibited more stable and less variable behavioral patterns under heat stress, whereas cows in the conventional parlor system showed higher panting levels and greater short-term fluctuations. These system-level differences directly address the study objective and support panting as a practical indicator for heat-stress monitoring in subtropical dairy systems.

## Simple Summary

Heat stress is a major challenge for dairy farming in subtropical regions, affecting cow welfare and productivity. This study compared how dairy cows respond to heat stress in an automated milking system (AMS) and a conventional milking system on a commercial farm in Taiwan. We found that panting was the most sensitive indicator, with cows in the conventional system showing approximately 50% higher panting levels than those in the AMS. Cows in the AMS also showed more stable patterns, characterized by lower variability in panting compared to the conventional system. These findings highlight the value of behavior-based monitoring for improving animal welfare and heat-stress management.

## 1. Introduction

Heat stress is one of the major environmental constraints limiting dairy production, animal welfare, and behavioral stability in tropical and subtropical regions [1,2,3]. In dairy cows, exposure to elevated temperature–humidity index (THI) disrupts thermoregulation, depresses feed intake, alters rumen function, and induces behavioral and physiological responses such as increased panting, reduced lying time, and changes in daily activity patterns [1,2,3]. Meta-analyses have consistently shown that milk yield, dry matter intake, and feed efficiency decline as THI increases [4,5]. Experimental studies indicate that dry matter intake may decrease by approximately 0.85 kg per day per °C increase in mean air temperature (2-day lag) [6], while milk yield declines by about 0.2 kg per cow per day per unit increase in THI above commonly reported threshold levels (≈72) [7] Modeling studies further demonstrate that the magnitude of production losses depends on both the intensity and duration of heat load [8].

Behavioral responses to heat stress, including panting, changes in feeding behavior, and alterations in rumination, have been widely used as sensitive indicators in dairy cows [9,10,11,12]. Panting responds rapidly to increasing THI, with respiration rate increasing substantially under heat stress conditions. Field-based studies have reported respiration rates averaging around 49–52 breaths/min under typical conditions, with increases of approximately 13–14 breaths/min per 10 °C rise in ambient temperature [13]. Respiration rate has been reported to increase by approximately 20–23 breaths/min under heat stress conditions compared with cooled cows, even under similar THI environments, highlighting the importance of heat abatement strategies [14]. In contrast, feeding and rumination typically exhibit more gradual or delayed changes due to reduced rumen motility and diminished feed intake under heat load [10,11,12]. Previous studies have also shown that moderate heat load alters grazing, standing, and shade-seeking behaviors in dairy cows, with shifts in time budgets characterized by decreased grazing and increased standing or shade-seeking behavior under elevated thermal conditions [15]. Collectively, these patterns support the use of behavior-based monitoring across production systems.

Milking-system environments may influence how heat-related behavioral responses are expressed. Automated milking systems (AMS) allow more flexible cow traffic, voluntary milking, and reduced handling stress compared with conventional milking systems [16], which may contribute to differences in behavioral organization under heat stress. In contrast, conventional parlor systems require fixed milking schedules and the congregation of cows in holding areas [17], conditions that may exacerbate thermal load and behavioral stress. Previous studies have shown that heat stress alters feeding, rumination, and activity patterns, with reported reductions in rumination time and milking frequency, as well as changes in daily activity and behavioral rhythms under elevated thermal load [18,19,20,21]. Monitoring systems in AMS environments have further demonstrated the ability to capture these behavioral changes in relation to thermal conditions.

AMS and conventional parlor systems differ in management structure, including milking frequency (events/day), cow traffic, and human–animal interaction. These differences may influence how cows respond to thermal stress under varying environmental conditions. Understanding differences in thermal responses across these milking systems requires analytical approaches that capture temporal dynamics (e.g., minutes/day of panting, feeding, and rumination) and variability patterns (e.g., day-to-day variation in behavior). Behavioral responses to heat stress may exhibit nonlinear and multivariate characteristics across THI gradients [22,23,24,25,26].

However, existing studies on heat stress and milking-system environments have primarily been conducted in temperate regions or under experimental conditions [2,3]. System-level comparisons of behavioral responses between AMS and conventional milking systems under comparable commercial farm conditions provide important insights into how management structure influences responses to thermal stress. In Taiwan, labor constraints and management challenges have contributed to the increasing adoption of AMS [27], offering an opportunity to examine how different milking systems shape behavioral responses under real-world subtropical conditions. Transitional farm configurations, where AMS and conventional systems coexist within the same facility, enable direct system-level comparisons under closely aligned environmental and management conditions. Furthermore, applying multivariate approaches allows integrated characterization of behavioral responses to heat stress, capturing coordinated patterns across multiple behavioral domains [25,26].

Accordingly, this study aimed to compare heat-related behavioral responses between milking systems under subtropical commercial conditions. We hypothesized that cows in the AMS would exhibit more temporally stable and less variable behavioral patterns under heat stress than those in a conventional parlor system. Using high-resolution behavioral time-series data, we evaluated temporal variation and multivariate behavioral responses of panting, feeding, and rumination in relation to thermal load.

## 2. Materials and Methods

### 2.1. Study Site and Experimental Period

The study was conducted from 18 August to 25 October 2025, on a commercial Holstein dairy farm in Taoyuan, Taiwan (24°58′44.0″ N, 121°02′10.3″ E), located in a subtropical region characterized by hot and humid summers. During the study period, the average ambient temperature was 29.2 °C (range: 22.6–31.4 °C), and the average relative humidity was 78.1%, based on local meteorological records. Daily ambient temperature, relative humidity, and temperature–humidity index (THI) were continuously recorded, and their temporal variation is presented in Supplementary Figure S1. This study was conducted as an observational, system-level field study without experimental manipulation or replication at the farm level. The barn was considered the primary unit of observation. No randomization or replication at the farm level was applied. This design limits causal inference but reflects real-world production conditions.

### 2.2. Barn Layout and General Management

The farm consisted of two adjacent free-stall barns sharing a central total mixed ration (TMR) feeding alley. Both barns were identical in structural design and dimensions (16 m in width and 54 m in length) and were configured as 3-row free-stall barns, with two rows arranged in a head-to-head configuration. Each barn was equipped with two scraper alleys measuring 4.2 m and 3.6 m in width, respectively. Prior to the study, both barns were originally equipped with 111 freestalls and housed approximately 70 lactating cows each.

Management conditions were identical between barns, including cooling systems, feeding management, space allowance/stocking density, and water access. All cows were initially milked using a conventional parlor system. Automated manure scrapers operated six times daily. Ventilation consisted of axial-flow fans activated at 28 °C and high-pressure misters activated at 30 °C and 75% relative humidity, installed along the feed alley and holding pen. All resting and feeding areas were fully shaded to eliminate direct solar radiation.

### 2.3. Milking Systems and Automated Milking Systems (AMS) Implementation

Following partial remodeling of one barn to accommodate an automated milking system (AMS; DeLaval VMS V300, DeLaval International AB, Tumba, Sweden), a designated corner of the facility was converted for AMS installation and cow-traffic infrastructure. As a result, the number of available freestalls in the AMS barn was reduced from 111 to 90.

Given the practical operating capacity of the AMS and to ensure stable cow traffic and adequate space allowance, 65 cows from the original barn population were retained and transitioned to the AMS and were designated as Group A, whereas the neighboring barn continued operating under the conventional parlor system and was designated as Group B. No random allocation of cows to treatments was performed, as grouping followed routine farm management practices.

Before formal data collection, the AMS barn underwent hardware installation and layout optimization, including the establishment of a guided-flow cow-traffic system following a milk-first pattern with a no-feed VMS operation. The traffic design connected the observation area, drinking zone, and milking unit to promote consistent unidirectional cow movement through the system.

### 2.4. AMS Adaptation and Data Collection Timeline

Operational milking in the AMS commenced on 4 August 2025, when cows completed their initial milking sessions. Between 4 and 9 August cows progressively adapted to the system, with an increasing proportion able to access and complete milking without assistance. Following structural modifications, cows were allowed an adaptation period of approximately two weeks to become familiar with voluntary movement through the smart selection gate and the designated AMS pathway.

To ensure stable AMS utilization and minimize behavioral variability associated with system familiarization, formal data collection for both AMS and conventionally milked cows began on 18 August 2025. Although behavioral data were available from 10 August onward, the early post-installation phase exhibited greater variability; therefore, all primary analyses presented in the main text were restricted to data collected after stabilization of cow traffic patterns and AMS use. To assess the robustness of the findings, supplementary analyses were conducted using data from the full observation period (10 August–25 October), including the AMS adaptation phase (Table S1).

All observations were conducted within the normal management routines of the commercial dairy farm and did not involve any invasive procedures. The study followed the principles of animal welfare and complied with Taiwan’s Animal Protection Act and standard dairy farm management guidelines.

### 2.5. Animals and Baseline Characteristics

A total of 140 clinically healthy multiparous Holstein cows were enrolled, comprising 65 cows in Group A and 75 cows in Group B. All cows were free of mastitis, lameness, and metabolic disorders at enrollment.

Baseline parity averaged 2.06 ± 1.12 in Group A and 1.75 ± 1.03 in Group B. Mean days in milk at baseline were 241.6 ± 133.9 d and 253.8 ± 127.6 d for Group A and B, respectively. Baseline production level was defined as the mean DHI test-day milk yield (kg/d) recorded once monthly during February–July 2025, representing the average of approximately six monthly test-day records per cow; corresponding values were 28.65 ± 4.34 kg/d for Group A and 26.76 ± 5.87 kg/d for Group B. These baseline summaries were used for descriptive contextualization only and were not intended for statistical inference or treatment comparison.

### 2.6. Feeding Management

All cows received the same TMR diet throughout the study period, containing 14.17% crude protein, 3.85% ether extract, 38.18% neutral detergent fiber, 35.45% non-fiber carbohydrates, and 21.32% starch. The ration was offered three times daily (05:00, 08:00, and 15:00), with feed refusals maintained <5% to ensure adequate intake. Clean drinking water was available ad libitum.

### 2.7. Environmental Monitoring and THI Calculation

Environmental monitoring was conducted using temperature–humidity sensors (model RS8005-uc, YoLink, Lake Forest, CA, USA), which were installed by Integrated Telecom Module & Co., Taichung, Taiwan, on barn walls approximately 60 cm above cow head height. These sensors recorded air temperature and relative humidity at 10 min intervals. All data were transmitted via encrypted networks and stored on a secure Amazon Web Services (AWS, Amazon.com, Inc., Seattle, WA, USA) cloud platform, enabling continuous remote verification of barn microclimate conditions.

The THI was calculated using the formula proposed by the National Research Council (NRC) as follows: THI = (1.8 × T + 32) − [(0.55 − 0.0055 × RH) × (1.8 × T − 26.8)], where T is ambient temperature (°C), and RH is relative humidity (%) [28].

### 2.8. Behavioral Monitoring and Sensor Validation

Behavioral data, including panting time, feeding time, and rumination time, were continuously recorded for all cows using Allflex^®^ Heatime^®^ Pro+ neck-collar systems (SCR Engineers Ltd., Netanya, Israel). The collars employ tri-axial accelerometer–based sensing to quantify time-budgeted behavioral activities and automatically generate summarized outputs at 2 h intervals, which were transmitted to the farm management server for subsequent analysis. The Heatime^®^ Pro system has been widely applied in dairy research [29,30].

Panting behavior was quantified as total daily panting duration (minutes). To ensure the reliability of collar-derived panting measurements, on-farm validation was conducted using synchronized visual observations on a randomly selected subset of approximately 10% of cows. Agreement between collar-derived measurements and visual observations was assessed as percentage agreement and exceeded 90%. In addition, a strong positive correlation was observed between the two methods (r = 0.88), indicating high consistency, consistent with previous validation studies [31].

### 2.9. Justification for Behavioral Indicators

Rectal temperature, respiration rate, and heart rate were not collected for methodological and practical reasons. Continuous physiological monitoring during peak summer months was not feasible under commercial farm conditions due to labor demands, equipment constraints, and concerns that repeated handling could exacerbate heat load. In addition, the Heatime^®^ Pro+ system is designed for behavioral sensing, and integrating additional physiological devices was incompatible with routine farm operations.

Accumulating evidence indicates that behavioral responses, particularly panting, reductions in feeding activity, and disruptions in rumination, are biologically meaningful and sensitive indicators of heat stress, with strong associations to physiological outcomes [1,31,32]. Therefore, behavioral indices were prioritized as continuous, minimally invasive, and scalable measures of thermal load under real-world production conditions.

### 2.10. Modelling

Daily panting, feeding, and rumination time-series data were analyzed descriptively at the barn level, with no attempt to infer causal differences between systems, to characterize temporal patterns and associations with short-term thermal load, defined as mean THI from 11:00 to 16:00 h. Linear regression models were used to summarize overall temporal trends and co-variation with thermal conditions. Monotonic temporal patterns were further characterized using the Mann–Kendall trend test.

Nonlinear behavioral trajectories were described using generalized additive models (GAM) fitted with a Gaussian error distribution. Smooth terms were specified as group-specific smooth functions of DayIndex using thin plate regression splines. Models were implemented using the mgcv package in R software (version 4.5.0; R Foundation for Statistical Computing, Vienna, Austria).

### 2.11. Statistical Analysis of Data

All statistical analyses were performed using R software (version 4.5.0; R Foundation for Statistical Computing, Vienna, Austria). Descriptive statistics, including means, standard deviations, and 95% confidence intervals (CI), were calculated to summarize behavioral distributions within each barn.

Statistical models were used to characterize temporal patterns and associations rather than to support hypothesis testing or inferential comparisons between milking systems. Associations between THI and behavioral outcomes were described using linear models fitted separately at the barn level, with THI and group terms included to summarize overall patterns of co-variation.

Short-term heatwave effects were examined by classifying days as normal or heatwave based on THI thresholds, followed by within-group paired contrasts to describe behavioral changes associated with heatwave exposure. Threshold-like responses were explored descriptively using piecewise (broken-stick) regression models based on 11:00–16:00 h THI.

Multivariate behavioral patterning under varying thermal conditions and barn-level environments was explored using principal component analysis (PCA) based on standardized panting, feeding, and rumination variables. PCA was applied as an exploratory tool to visualize coordinated behavioral variation rather than for inferential hypothesis testing.

All statistical analyses were interpreted as descriptive and exploratory, consistent with the non-replicated, system-level study design.

## 3. Results

### 3.1. Temporal Dynamics and Smoothed Behavioral Trends of Panting, Feeding, and Rumination in Cows from Group A and Group B

#### 3.1.1. Panting-Time Difference over Time

As summarized in Table 1, panting-time differences (Panting_A − Panting_B) showed a significant increasing trend over time (β = 0.65 ± 0.11 min/day, t = 6.21, *p* < 0.001), indicating a gradual reduction in the magnitude of between-group differences. At the start of the observation period, panting duration in Group A was substantially lower than in Group B (−55.73 min), and although this gap narrowed over time, it remained negative throughout the study. Including midday temperature–humidity index (THI) (11:00–16:00) as a covariate did not materially alter the temporal trend (β = 0.04 ± 0.63), indicating that short-term fluctuations in thermal conditions did not substantially explain the observed convergence in panting-time differences. This suggests that, within the temporal scale of this analysis, panting responses may be more strongly influenced by cumulative heat load and system-level management factors, rather than immediate environmental variation, although THI remains a relevant indicator of general heat stress conditions. The model explained approximately 35–36% of the variance (adjusted R2 ≈ 0.35), indicating a moderate temporal structure in the data (Table 1).

#### 3.1.2. Synchrony of Panting Behavior Between Groups

Cross-correlation analysis showed the strongest association at lag 0, exceeding the 95% confidence band, indicating close alignment of day-to-day fluctuations in panting duration between groups. Moderate positive correlations were also observed at short lags (±1 to ±2 days), suggesting parallel short-term variation.

No consistent leading or lagging pattern was observed across lags. Overall, the two time series exhibited similar temporal structure, with concurrent rises and declines, consistent with shared responses to common thermal conditions, as reflected by THI, rather than asynchronous dynamics between groups (Supplementary Figure S2).

#### 3.1.3. Monotonic Trends in Panting Behavior

Mann–Kendall tests indicated negative monotonic trends in panting duration for both groups over the observation period (Table 2). Group A showed τ = −0.26 (*p* = 0.0017), while Group B showed a stronger negative trend (τ = −0.46, *p* < 0.001), indicating a consistent decline in panting time in both groups.

#### 3.1.4. Multi-Behavioral Patterns (Panting, Feeding, and Rumination)

**Figure 1:**
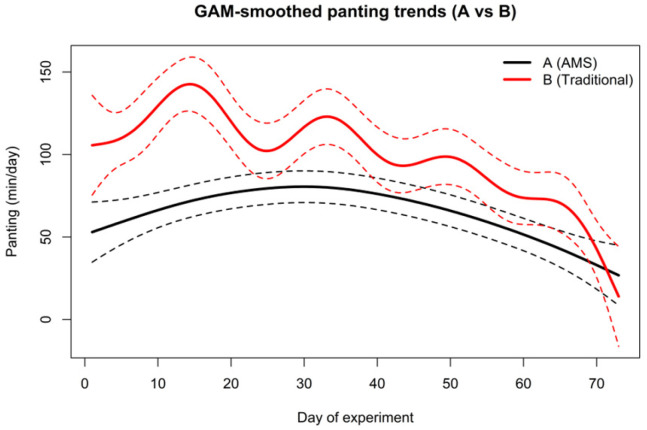
GAM-smoothed panting time series for Group A (AMS) and Group B (traditional) over the observation period. Solid lines represent smoothed mean trajectories, and dashed lines indicate 95% confidence intervals.

**Figure 2:**
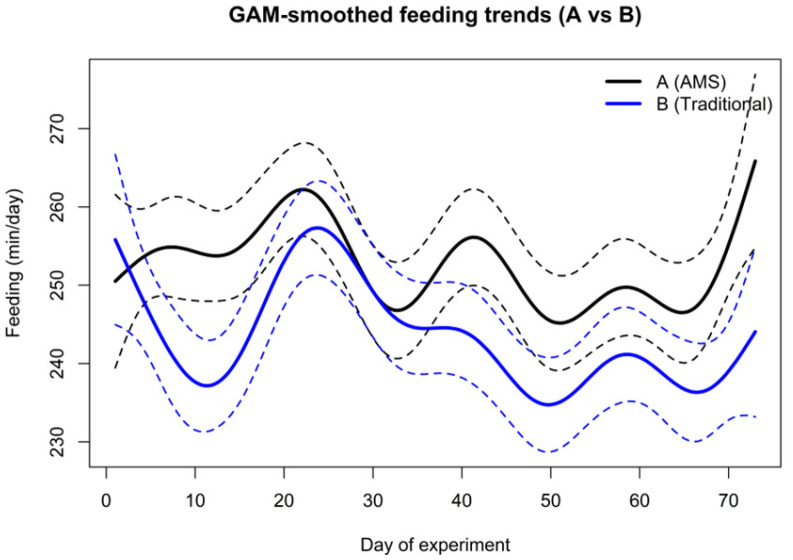
GAM-smoothed feeding time series for Group A (AMS) and Group B (traditional). Solid lines represent mean trajectories, and dashed lines indicate 95% confidence intervals.

**Figure 3:**
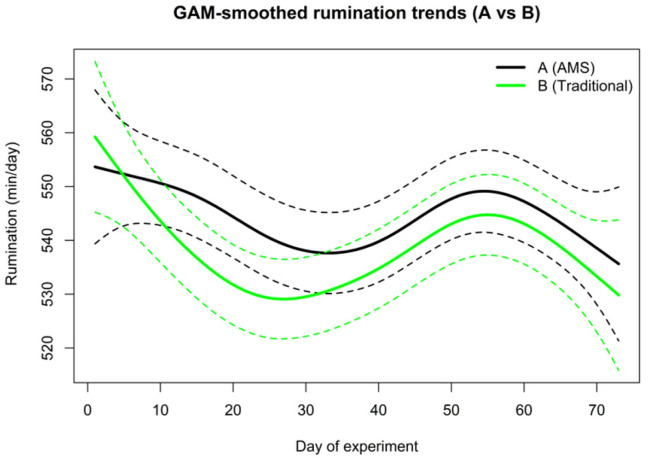
GAM-smoothed rumination time series for Group A (AMS) and Group B (traditional). Solid lines represent mean trajectories, and dashed lines indicate 95% confidence intervals.

Panting exhibited the largest between-group difference, with Group B showing higher average values by approximately 33 min/day and greater short-term variability. In contrast, Group A displayed smoother temporal dynamics with lower fluctuation (Figure 1). Feeding differences were smaller in magnitude, with Group B showing a modest reduction of approximately 9.5 min/day relative to Group A, while both groups exhibited comparable short-term variability (Figure 2). Rumination showed the smallest between-group difference (approximately 6.8 min/day), with low variability and weak temporal structure in both groups, indicating limited sensitivity to short-term environmental variation (Figure 3). Model performance further supported these patterns, with panting explaining the greatest proportion of temporal variation (adjusted R^2^ = 0.56), followed by feeding (0.40), whereas rumination showed substantially lower explanatory power (0.16) (Table 3).

### 3.2. Behavioral Variability and Descriptive Summaries Across Milking-System Environments

Panting exhibited greater variability in the traditionally milked barn (SD = 37.7 min/day) compared with the AMS barn (SD = 25.9 min/day), corresponding to an approximately 45% higher dispersion. Although coefficients of variation were similar between groups (≈38–39%), the wider confidence interval in Group B (90.5–108.4 min/day) indicates greater day-to-day fluctuation at the system level. In contrast, feeding and rumination showed relatively low variability in both systems (CV ≈ 3–4%), with largely overlapping confidence intervals, indicating more stable behavioral patterns and limited differentiation between barns for these behaviors (Table 4).

### 3.3. Heat Sensitivity and THI–Group Interaction Effects on Behavioral Responses

To describe associations between thermal load and behavioral responses within each barn (Group A and Group B), linear regression models were fitted, including THI as a predictor for panting, feeding, and rumination behaviors. Interaction terms between THI and group were included for descriptive purposes and did not indicate clear divergence in THI-associated behavioral patterns between barns (Table 5).

Across behaviors, differences in THI-associated slopes were observed. Panting increased with rising THI in both barns, with a numerically steeper slope in Group B compared with Group A (5.88 vs. 4.26 min per unit THI), representing an approximately 38% greater response magnitude. Feeding behavior showed minimal and inconsistent responses, with a slight decrease in Group A (−0.25 min per unit THI) and a slight increase in Group B (+0.21 min per unit THI). These differences were small in magnitude and did not indicate a clear divergence in feeding responses between systems. The lower feeding duration observed in Group A at the descriptive level should therefore be interpreted cautiously and does not necessarily reflect reduced feeding performance under thermal stress. Given that both barns received identical feeding schedules within the same housing structure, these patterns are unlikely to be explained by differences in feeding management. Instead, they may reflect differences in behavioral organization associated with milking-system design. Specifically, variation in cow traffic patterns, activity synchronization, and spatial distribution may influence how feeding behavior is temporally expressed under thermal load, rather than the overall feeding capacity.

### 3.4. Behavioral Adjustments to Heatwave Conditions Across Milking-System Environments

Panting showed the largest numerical change during heatwave periods (Table 6). In the AMS barn (Group A), average daily panting duration increased from 44.5 to 73.3 min/day (approximately +65%), whereas in the traditionally milked barn (Group B), panting increased from 74.1 to 108.3 min/day (approximately +46%). Despite these increases, absolute panting duration remained consistently higher in Group B across the observation period.

Feeding behavior showed minimal changes during heatwave exposure. In the AMS barn, average daily feeding time decreased slightly (256.0 to 251.0 min/day; approximately −2%), whereas feeding duration in the traditionally milked barn remained largely stable (241.0 to 243.0 min/day; <1% change). These results indicate limited short-term sensitivity of feeding behavior to acute heatwave conditions at the barn level.

Rumination exhibited the smallest apparent response to heatwave conditions. In the AMS barn, rumination time increased slightly (537.0 to 547.0 min/day; approximately +2%), while rumination in the traditionally milked barn remained stable (536.0 to 538.0 min/day; <1% change). Compared with panting and feeding, rumination appeared less responsive to short-term heatwave exposure in both systems.

Overall, these results indicate that heatwave conditions primarily affected panting behavior, with comparatively minor adjustments observed in feeding and rumination. These findings describe shifts in behavioral time budgets within each barn-level system.

### 3.5. Cross-Correlation Between Thermal Load and Behavioral Responses

Panting exhibited a strong and consistent temporal association with thermal load in both systems, representing one of the clearest behavioral responses to short-term heat stress. The peak cross-correlation occurred at a lag of −1 day in both barns (Group A: r = 0.67; Group B: r = 0.68), indicating a strong association and that day-to-day fluctuations in panting closely tracked changes in THI with an approximately one-day lead (Table 7). The similar magnitude of correlation coefficients across systems suggests comparable sensitivity of panting responses to thermal variation.

In contrast, rumination showed substantially weaker and delayed associations with THI. In the AMS barn, the strongest association occurred at a lag of −1 day (r = 0.18), whereas in the traditionally milked barn, peak associations occurred at a lag of −2 days (r = 0.17), indicating a more gradual and temporally shifted response to thermal load. The markedly lower correlation coefficients (approximately 3–4 times smaller than those for panting) further indicate reduced sensitivity of rumination to short-term thermal fluctuations.

Overall, these results demonstrate clear differences in temporal response dynamics across behaviors. Panting exhibited rapid and tightly coupled responses to short-term thermal variation, whereas rumination showed weaker, delayed, and less synchronized adjustments. These patterns support the use of panting as a sensitive and near-real-time behavioral indicator of heat stress under subtropical conditions.

### 3.6. THI (11:00–16:00) Breakpoint Detection and Behavioral Slope Patterns

Using the average THI during the peak heat-load window (11:00–16:00), broken-stick models were fitted descriptively to characterize potential changes in THI–behavior slopes within each barn-level system (Table 8). Estimated breakpoints varied across behaviors, ranging from THI 74.29 to 87.06, with panting showing the largest numerical changes in slope magnitude.

For panting behavior, broken-stick models identified an estimated breakpoint at approximately THI 87 in both barns. While this relatively high breakpoint likely reflects the distribution of observed data under subtropical summer conditions, it also indicates that rapid escalation in panting responses occurred primarily under extreme thermal load. Below the breakpoint, panting increased at moderate rates (Group A: +4.15; Group B: +5.62 min per unit THI), whereas above the breakpoint, slope estimates increased substantially (Group A: +25.55; Group B: +56.65), representing an approximately 6-fold increase in Group A and a 10-fold increase in Group B. These patterns indicate a nonlinear acceleration in panting response at high THI levels, with a stronger response magnitude observed in the traditionally milked barn. However, slope estimates beyond the breakpoint were associated with considerable uncertainty and should be interpreted cautiously.

Feeding behavior exhibited weak and inconsistent associations with THI. In the AMS barn, the estimated slope shifted from slightly negative below the breakpoint (−0.48) to slightly positive above it (+1.50), whereas in the traditionally milked barn, slope estimates remained small across the breakpoint (+0.28 to −1.03), indicating minimal sensitivity to increasing thermal load.

Rumination also showed weak and heterogeneous associations with THI. In the AMS barn, the slope changed from negative below the breakpoint (−10.92) to positive above it (+2.16), whereas in the traditionally milked barn, slope estimates remained small across the breakpoint (+0.64 to −5.12), suggesting limited and inconsistent responses to thermal variation.

Overall, these results demonstrate that panting exhibits a threshold-dependent and rapidly escalating response to high THI, whereas feeding and rumination show weaker, more variable, and less consistent patterns. Breakpoint estimates should be interpreted cautiously due to the limited number of observations under extreme THI conditions.

### 3.7. Multivariate Behavioral Differentiation Under Barn Management and Heat-Load Conditions

**Figure 4:**
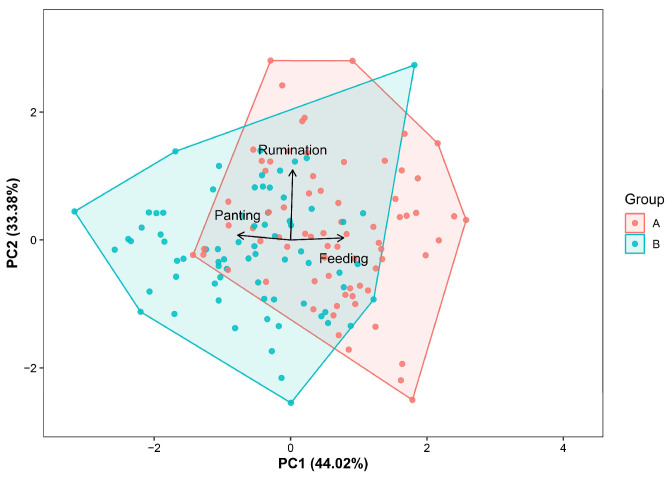
PCA of daily panting, feeding, and rumination behaviors in Group A (AMS) and Group B (traditional). Points represent daily observations, and polygons indicate the 95% data hull for each group.

PCA was used to summarize the multivariate structure of daily panting, feeding, and rumination behaviors at the barn level (Figure 4). The first two principal components explained 43.35% (PC1) and 33.09% (PC2) of the total variance. Behavioral distributions from Group A and Group B showed substantial overlap, indicating broadly similar multivariate organization of the monitored behaviors. Nevertheless, differences in dispersion were evident, with observations from Group A forming a more compact cluster and those from Group B occupying a broader region of the PCA space, indicating greater spread of observations at the system level in Group B.

**Figure 5:**
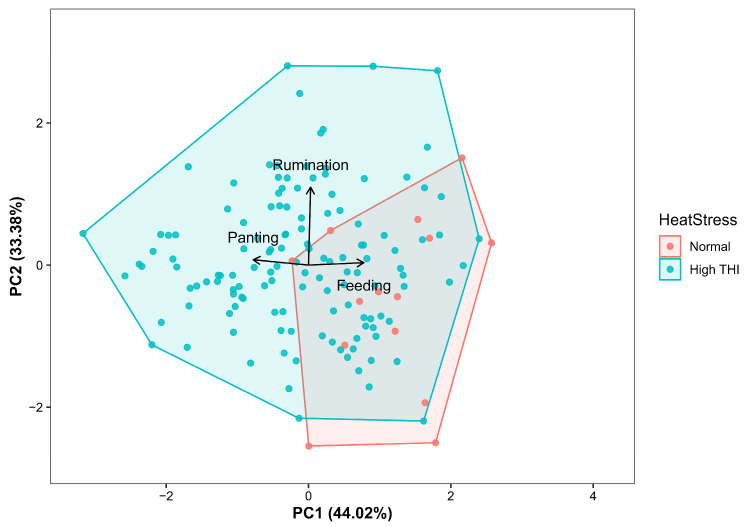
PCA of daily panting, feeding, and rumination behaviors under normal and high THI conditions. Points represent daily observations, and polygons indicate the 95% data hull for each condition.

Behavioral loadings indicated that panting and feeding contributed most strongly to variation along PC1, with panting loading positively and feeding loading negatively, whereas rumination loaded primarily along PC2. These loading patterns suggest that the three behaviors jointly structured the dominant multivariate gradients observed across days. The more compact clustering observed in Group A indicates greater behavioral consistency, whereas the broader dispersion in Group B reflects increased variability in behavioral organization under similar environmental conditions. To further examine the influence of thermal conditions on multivariate behavioral organization, PCA was repeated after classifying days into normal and high-THI conditions (Figure 5). In this analysis, PC1 and PC2 again explained 43.35% and 33.09% of the total variance. Compared with normal-THI days, high-THI days occupied a broader and more dispersed region of the PCA space, indicating increased variability in behavioral patterns under elevated thermal load. In contrast, normal-THI days formed more compact clusters, reflecting more stable behavioral organization. Loading directions remained consistent with those observed in Figure 4, suggesting that the underlying behavioral structure was preserved across thermal conditions, while the degree of dispersion varied.

## 4. Discussion

**Figure 6:**
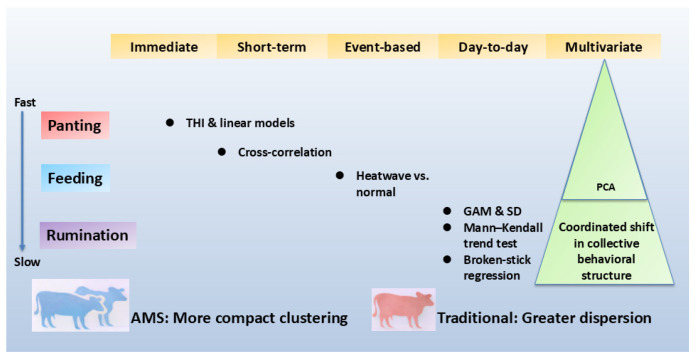
Conceptual framework illustrating the temporal organization and analytical integration of behavioral responses to heat stress in dairy cows managed under AMS and traditional parlor systems. Panting, feeding, and rumination are positioned according to their relative response speed to increasing thermal load (from fast to slow) and the temporal scales at which they were examined, including immediate and short-term associations, event-based contrasts during heatwaves, day-to-day trends and threshold-based patterns, and multivariate integration. Analytical approaches applied at each temporal scale are indicated schematically, including THI-based linear models, cross-correlation analysis, heatwave contrasts, generalized additive models, Mann–Kendall trend tests, broken-stick regression, and PCA. The framework summarizes how panting consistently emerged as a rapidly responding and THI-sensitive behavior across analytical scales, whereas feeding and rumination reflected slower, cumulative, and system-modulated responses. Arrow direction denotes conceptual progression across temporal and analytical scales; arrow length and thickness are schematic and do not represent quantitative effect sizes. This figure is intended as a descriptive synthesis of analytical findings rather than an inferential comparison between milking systems.

This study provides an integrated, descriptive evaluation of how dairy cows housed in an AMS barn and a traditional parlor barn expressed panting, feeding, and rumination behaviors across multiple temporal scales of thermal challenge in a subtropical environment (Figure 6). By combining time-series visualization, variability metrics, heatwave contrasts, THI–behavior associations, breakpoint exploration, and multivariate profiling, we illustrate that management-associated patterns were reflected in the observed magnitude, temporal structure, and coordination of behavioral responses at the system-level, without implying causal or inferential comparisons between systems.

Panting likely reflects an immediate thermoregulatory response to increased heat load, driven by increased respiratory evaporative heat loss to maintain core body temperature [1,13,14,31]. In contrast, feeding and rumination responses are generally more gradual and cumulative, likely reflecting changes in rumen activity, metabolic heat production, and energy balance under sustained heat exposure [10,12]. The more temporally stable behavioral patterns observed in the AMS barn may be associated with management-related factors, including more distributed cow traffic, reduced congregation, and lower handling stress. Conversely, greater short-term fluctuation in the traditional parlor barn may reflect synchronized cow movement, pre-milking gathering, and localized heat accumulation. Together, these mechanisms suggest that both physiological regulation and management structure contribute to the organization of behavioral responses under subtropical heat stress. These mechanisms are supported by previous studies showing that cow traffic, social hierarchy, and access to milking systems influence spatial and temporal patterns of behavior, particularly in AMS environments where voluntary movement reduces crowding and facilitates behavioral thermoregulation [17,18,19,20,33].

Importantly, this study highlights that heat-stress responses are structured at the system level, with differences emerging in temporal dynamics, variability, and behavioral coordination rather than solely in mean response levels. This system-level perspective provides a complementary framework to conventional individual-based analyses and may improve the interpretation of behavioral indicators under commercial production conditions.

Over the study period, both barns showed declining trends in daily panting duration, consistent with seasonal reductions in ambient thermal load. However, the traditional parlor barn exhibited steeper monotonic trends and greater short-term oscillations, as reflected by wider CI in GAM-smoothed trajectories. These temporal features may reflect heightened behavioral responsiveness to sustained thermal conditions rather than smooth adaptive stabilization. In contrast, the AMS barn maintained consistently lower observed panting levels with attenuated short-term variability, suggesting relatively greater behavioral stability at the system level during prolonged heat exposure. Such patterns may reflect system characteristics commonly associated with AMS environments, including voluntary milking, reduced handling frequency, and more autonomous access to feed and resting areas, though causal attribution cannot be established within the present observational design.

Temporal divergence between systems was further described using linear regression summaries of the intergroup panting difference (Panting_A − Panting_B) over the observation period. The panting-time difference showed a positive temporal trend, becoming progressively less negative across days, whereas short-term THI (11:00–16:00) did not account for additional variation in the difference. These observed patterns indicate that temporal changes in panting differences were more closely aligned with cumulative system-level dynamics rather than short-term thermal fluctuations. Despite differences in variability and magnitude, panting responses in both systems exhibited highly similar temporal associations with thermal load. Cross-correlation analyses showed strong positive associations between THI and panting at a lag of −1 day in both AMS and traditionally milked cows, indicating close temporal coupling between changes in thermal conditions and panting behavior. Autocorrelation analyses further revealed strong day-to-day similarity but limited long-term persistence, consistent with panting functioning as a short-term THI-responsive indicator of acute heat exposure.

Behavioral stability varied between milking-system environments across multiple descriptive metrics. The AMS barn was characterized by narrower CI in GAM-smoothed trajectories and more compact clustering in PCA space, reflecting lower observed short-term variability in panting, feeding, and rumination behaviors [16,33]. Descriptive summaries of daily behavioral distributions and variability metrics were consistent with these patterns, showing lower standard deviations in the AMS barn, particularly for panting behavior. In contrast, the traditional parlor barn exhibited broader variability and greater dispersion, especially under elevated thermal load. These patterns further support the role of system-level factors in shaping behavioral responses. While panting is widely recognized as a rapid and sensitive indicator of heat stress [9,16], the present results highlight that differences in temporal stability and variability may provide additional insight into heat-stress responses beyond absolute behavioral levels. Under prolonged exposure to high temperature and humidity, as commonly observed in subtropical dairy systems, management-related factors such as cow movement, congregation patterns, and environmental exposure may play a more prominent role in shaping behavioral organization. This system-level perspective may help explain why differences in behavioral stability were observed between milking environments even when overall responses to thermal load appeared similar.

Associations between THI and behavioral responses further support these patterns by indicating differences in response magnitude rather than response direction. Although THI × group interaction terms were not statistically significant, slope estimates showed a numerically steeper increase in panting duration per unit rise in THI in traditionally milked cows. These patterns suggest that, while both systems respond similarly to increasing thermal load, the intensity of response may differ. In contrast, feeding and rumination behaviors exhibited weak and inconsistent associations with THI in both systems, consistent with previous observations that these behaviors respond more gradually or nonlinearly to thermal load and may reflect accumulated rather than immediate heat exposure [12,15].

Nonlinear analyses further highlighted panting as the most responsive behavioral indicator of acute thermal challenge. Broken-stick regression models yielded similar estimated THI breakpoints (approximately 87) in both systems, beyond which panting showed numerically steeper increases, particularly in traditionally milked cows. Although slope estimates above the breakpoint were imprecise and did not reach statistical significance, the observed numerical patterns describe a tendency toward greater escalation of panting at higher THI levels in conventionally managed systems. In contrast, feeding and rumination exhibited weaker and less consistent breakpoint-related changes, indicating lower immediate sensitivity to abrupt increases in thermal load and more gradual adjustment patterns under sustained heat stress.

Event-based comparisons were consistent with these observed patterns. Heatwave periods were associated with marked increases in panting duration in both systems, with numerically larger increases observed in traditionally milked cows. Feeding and rumination showed minimal or delayed changes during heatwave episodes, supporting earlier evidence that panting responds rapidly to acute thermal challenge, whereas other behaviors integrate thermal stress over longer temporal scales [9,12,16].

At the multivariate level, PCA illustrated differences in multivariate behavioral configurations associated with milking-system environments and thermal conditions, as reflected by contrasts between the AMS barn and the traditional parlor barn, as well as between normal and high-THI days. These results describe coordinated variation across multiple behavioral domains, rather than isolated changes in individual behaviors, under increasing thermal load, highlighting the importance of multivariate behavioral integration in heat-stress assessment [1,25].

Comparable multivariate differentiation in response to heat stress has been reported previously, underscoring the value of integrative behavioral profiling for characterizing system-level responses to thermal challenge [18,19,25]. In the present study, the AMS barn was characterized by more compact clustering in PCA space, whereas high-THI days were associated with broader dispersion, reflecting increased multivariate variability under elevated thermal conditions.

Taken together, these findings describe associations between milking-system environments and the organization of behavioral responses under subtropical heat stress, without implying causal effects. Reduced dispersion in multivariate behavioral patterns and more consistent panting dynamics in the AMS barn suggest relatively greater behavioral coherence at the system level. These observations support the utility of panting behavior as a robust component of multivariate heat-stress monitoring frameworks and highlight its practical relevance for automated surveillance applications in environments where continuous behavioral data are available [32].

From a practical perspective, panting behavior may serve as a real-time and non-invasive indicator for heat-stress monitoring in dairy operations. The strong and consistent association between panting and THI suggests that panting-based thresholds could be integrated into precision livestock monitoring systems to support early detection of heat stress. In AMS environments, where continuous behavioral data are available, such indicators may facilitate timely management interventions, including adjustments in ventilation, cooling strategies, or cow traffic management to mitigate heat load and improve animal welfare.

Several limitations should be acknowledged. This study was conducted within a single climatic context and was based on observations from a single farm with two adjacent barn systems, which may limit the generalizability of the findings. Although this design enabled controlled comparisons under highly consistent management and environmental conditions, thereby facilitating clearer system-level behavioral characterization, it does not support causal inference and reflects real-world production conditions. Potential baseline differences between groups, including parity, days in milk (DIM), and production level, were not experimentally controlled and may confound behavioral comparisons between milking systems. These factors should be considered when interpreting the results.

This relatively focused sample reflects the study’s aim of providing a controlled, system-level comparison rather than population-level inference. While this design enhances internal consistency and reduces environmental and management variability, it may limit the generalizability of the findings. Future research should expand to multi-farm or nationwide datasets in Taiwan to validate these patterns across diverse herd structures, management systems, and climatic conditions.

## 5. Conclusions

This study demonstrates that heat-stress responses in dairy cows are structured at the system level under subtropical commercial conditions, with differences emerging in temporal dynamics, variability, and behavioral coordination rather than solely in response magnitude. Panting consistently functioned as a rapid and sensitive indicator of thermal load, whereas feeding and rumination reflected more gradual and cumulative adjustments. The more temporally stable behavioral patterns observed in the AMS environment suggest that management structure can influence behavioral organization under heat stress. These findings highlight the value of behavior-based monitoring, particularly panting, as a practical and real-time indicator for precision livestock management. Future research should extend these findings using multi-farm datasets and integrate behavioral, physiological, and genetic indicators to further characterize heat-stress resilience across production systems.

## Figures and Tables

**Table 1:** Linear regression models for daily panting-time difference (Group A − Group B). Model 1 includes DayIndex only. Model 2 additionally includes THI (11:00–16:00). DayIndex represents the linear temporal trend across the observation period. THI refers to the average temperature–humidity index during the peak heat-load period.

	Predictor	Estimate Trend (β)	Std. Error
Model 1			
	Intercept	−55.73	4.15
	DayIndex	0.65	0.11
Model fit	R^2^ = 0.3686	Adjusted R^2^ = 0.36	
Model 2			
	Intercept	−59.01	55.45
	DayIndex	0.65	0.12
	THI (11:00–16:00)	0.04	0.63
Model fit	R^2^ = 0.37	Adjusted R^2^ = 0.35	

**Table 2:** Mann–Kendall trend results for daily panting behavior in Group A and Group B. Kendall’s τ indicates the direction and strength of monotonic temporal trends within each group.

Group	Tau	Trend Direction
A	–0.26	Decreasing
B	–0.46	Strong decreasing

**Table 3:** Generalized additive models (GAM) results for barn-level temporal patterns in panting, feeding, and rumination. GAMs were fitted separately for each barn. Coefficients represent differences in mean behavioral levels between groups. Effective degrees of freedom (edf) describe the complexity of temporal patterns. Adj. R^2^ = adjusted R^2^; Dev. explained = deviance explained.

Behavior	Group B Coefficient (±SE)	Edf (Group A)	Edf (Group B)	Adj. R^2^	Dev. Explained
Panting	33.32 ± 4.11	2.49	8.14	0.56	60.0%
Feeding	–9.53 ± 1.49	8.23	7.97	0.40	47.3%
Rumination	–6.81 ± 2.72	3.94	3.72	0.16	21.7%

**Table 4:** Descriptive statistics of daily panting, feeding, and rumination behaviors at the barn level. Values represent mean (min/day), standard deviation (SD), coefficient of variation (CV), and 95% confidence intervals (CI) for each behavior within each group.

Behavior	Group	Mean (min/day)	SD	CV (%)	95% CI (Lower)	95% CI (Upper)
Panting	A	66.1	25.9	39.2	59.9	72.2
Panting	B	99.4	37.7	37.9	90.5	108.4
Feeding	A	252.3	10.3	4.1	250.0	254.8
Feeding	B	243.2	10.0	4.1	240.4	245.1
Rumination	A	544.1	19.0	3.5	539.6	548.7
Rumination	B	537.3	14.8	2.8	533.8	540.9

**Table 5:** Linear model slopes describing associations between THI and behavioral responses in Group A and Group B. Slopes represent the change in behavior (min/day) per unit increase in THI. Models were fitted separately within each barn. Adjusted R^2^ indicates model fit.

Behavior	THI–Behavior Slope (Group A)	THI–Behavior Slope (Group B)	Adjusted R^2^
Panting	4.26 min/unit	5.88 min/unit	0.48
Feeding	–0.25 min/unit	+0.21 min/unit	0.17
Rumination	+0.82 min/unit	+0.60 min/unit	0.04

**Table 6:** Behavioral responses during normal and heatwave periods in Group A and Group B. Values represent mean (min/day) and standard deviation (SD) for normal and heatwave periods within each group.

Behavior	Group	Normal Mean (min/day)	Heatwave Mean (min/day)	SD (Normal/Heatwave)
Panting	A	44.5	73.3	25.3/21.9
	B	74.1	108.3	40.6/32.9
Feeding	A	256.0	251.0	5.38/11.3
	B	241.0	243.0	6.43/10.9
Rumination	A	537.0	547.0	21.5/17.7
	B	536.0	538.0	16.8/14.3

**Table 7:** Peak cross-correlation lag and strength (r_max) between THI and behavioral responses in Group A and Group B. Lag indicates the time shift (days) at which the maximum correlation occurs. r_max represents the peak cross-correlation coefficient.

Behavior	Group	Lag (Peak)	r_max
Panting	A	−1	0.67
	B	−1	0.68
Rumination	A	−1	0.18
	B	−2	0.17

**Table 8:** Broken-stick regression results for THI–behavior relationships in Group A and Group B. Breakpoints indicate estimated THI values where slope changes occur. Slopes represent changes in behavior (min/day) per unit increase in THI before and after the breakpoint. Estimates at high THI should be interpreted cautiously due to limited observations.

Behavior	Group	Breakpoint THI	Slope Before	Slope After
Panting	A	87.06	4.15	25.55
	B	87.06	5.62	56.65
Feeding	A	84.85	−0.48	1.5
	B	85.74	0.28	−1.03
Rumination	A	74.29	−10.92	2.16
	B	86.96	0.64	−5.12

## Data Availability

The data presented in this study are available on request from the corresponding author. The data are not publicly available due to institutional data protection policies and agreements with participating farms.

## Supplementary Materials

The following supporting information can be downloaded at: https://www.mdpi.com/article/10.3390/ani16111665/s1. Figure S1: Temporal variation in ambient temperature, relative humidity, and THI during the study period. Figure S2: Autocorrelation function (ACF) of daily panting time in AMS and conventional parlor barns under subtropical conditions. Table S1: Sensitivity analysis of broken-stick regression describing THI–behavior relationships in dairy cows under subtropical conditions.

## Abbreviations

The following abbreviations are used in this manuscript:

ACF	Autocorrelation Function
AMS	Automated Milking System
CI	Confidence Intervals
CV	Coefficient of variation
EDF	Effective Degrees of Freedom
GAM	Generalized Additive Models
PCA	Principal Component Analysis
SD	Standard Deviation
THI	Temperature–Humidity Index
TMR	Total Mixed Ration
