# Supplementary material for: Behavioral Indicators of Heat Stress in Dairy Cows Under Subtropical Conditions: Comparison of Milking Systems
Source: Animals (Basel). 2026 May 29;16(11):1665. doi: 10.3390/ani16111665 (PMC13255648; doi:10.3390/ani16111665)
Supplement: Supplementary file 1 [file animals-16-01665-s001.zip › animals-4271557-supplementary.pdf]

Manuscript title

Behavioral indicators of heat stress in dairy cows under subtropical conditions: comparison of milking systems

Authors

C.H. Chao[1a*], K.C. Hsu[2a], Y.C. Fan[3], C.H. Chi[2*]

Journal

Animals

Supplementary Table S1. Sensitivity analysis of broken-stick regression describing THI–behavior relationships in dairy cows under subtropical conditions.

Sensitivity analysis of broken-stick regression results describing associations between panting, feeding, and rumination behaviors and average THI during the peak heat-load window (11:00–16:00) across the full observation period (August 10–October 25, 2025), including the AMS adaptation phase. Broken-stick models were fitted descriptively at the barn level within each group separately to assess the robustness of estimated breakpoints and slope patterns to the inclusion of early observational periods. Results are presented to evaluate the stability of within-group THI–behavior relationships under alternative temporal definitions and are not intended to support inferential comparisons between milking systems or to define system-specific thermal thresholds.

Behavior	Group	Breakpoint THI	Slope Before	Slope After
Panting	A	80.59	5.65	2.20
	B	87.07	5.60	57.31
Feeding	A	84.85	−0.64	1.59
	B	86.49	0.24	−2.06
Rumination	A	74.29	−9.49	1.74
	B	83.94	0.86	−0.26

Supplementary Figure S1

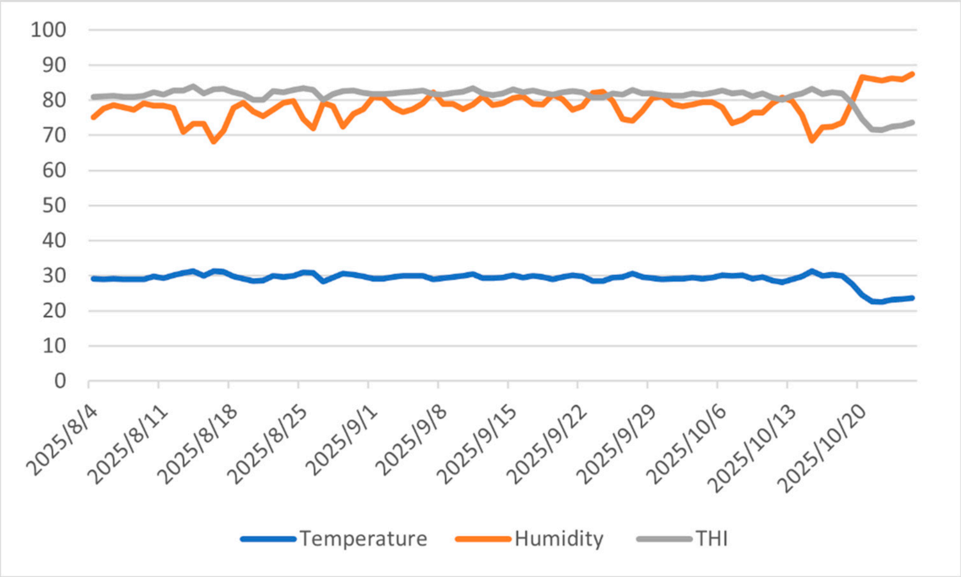

Supplementary Figure S1. **Temporal variation in ambient temperature, relative humidity, and THI during the study period**. Daily variation in ambient temperature (°C), relative humidity (%), and temperature–humidity index (THI) during the study period (August 4 to October 25, 2025) at the study site in Taoyuan, Taiwan. Values represent daily averages.

Supplementary Figure S2

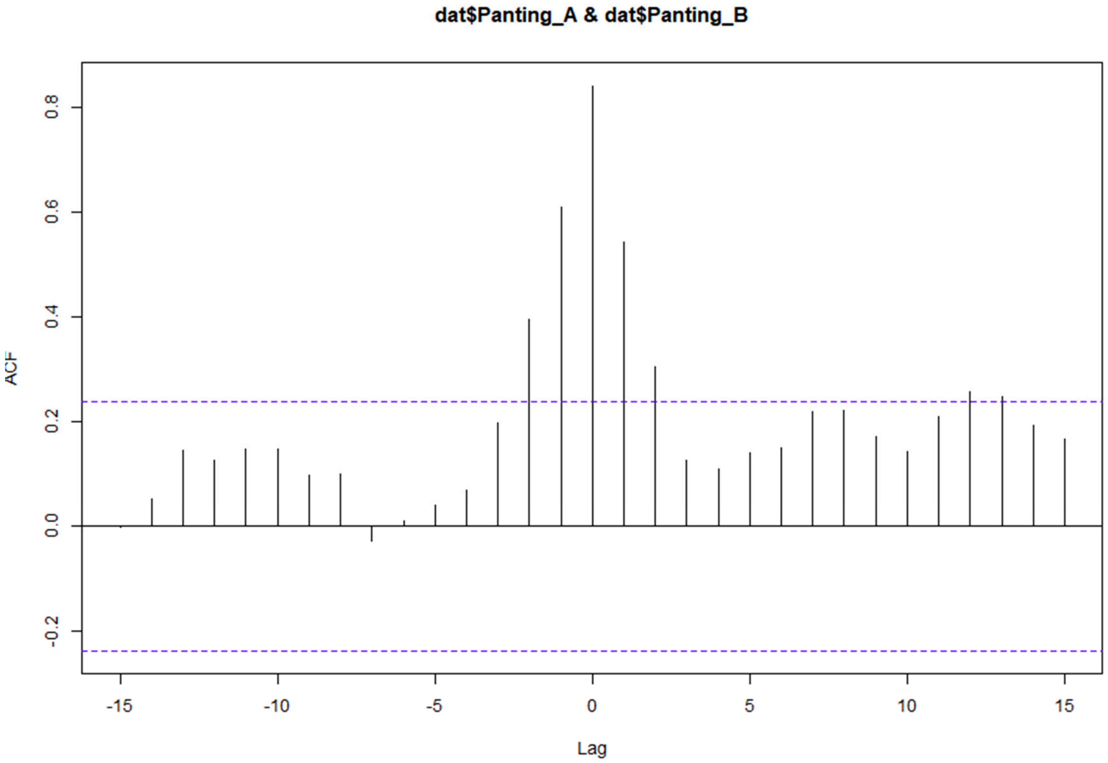

Supplementary Figure S2 Autocorrelation function (ACF) of daily panting time in AMS and conventional parlor barns under subtropical conditions.

Autocorrelation function (ACF) of daily panting time for the AMS barn (Panting_A) and the traditional parlor barn (Panting_B), analyzed jointly to characterize the shared temporal structure of panting behavior under common environmental conditions rather than to compare systems. The ACF showed a strong positive autocorrelation at lag 0, indicating pronounced day-to-day temporal dependence in panting duration across the dataset. Moderate positive autocorrelations were observed at lags 1–5, reflecting short-term temporal dependence likely associated with gradual changes in environmental heat load over consecutive days. Beyond approximately ±6 lags, most autocorrelation coefficients fell within the 95% confidence bounds, suggesting limited long-term persistence in panting patterns. Overall, these results indicate that panting behavior was influenced by slowly varying environmental conditions—such as multi-day heat events—without evidence of extended cyclic or strongly periodic structure.
